# Docking analysis of importin-11 homology model with the phyto compounds towards colorectal cancer treatment

**DOI:** 10.6026/97320630016153

**Published:** 2020-02-29

**Authors:** Jayaraman Selvaraj, Rajagopal Ponnulakshmi, Srinivasan Abilasha, Devarajan Nalini, Periyasamy Vijayalakshmi, Veeraraghavan Vishnupriya, Surapaneni Krishna Mohan

**Affiliations:** 1 Department of Biochemistry, Saveetha Dental College and Hospitals, Saveetha Institute of Medical and Technical Sciences, Saveetha University, Chennai - 600 077, India; 2 Central Research Laboratory, Meenakshi Academy of Higher Education and Research (Deemed to be University), Chennai-600 078, India; 3 Department of Anatomy, Asan Memorial Dental College & Hospital, Asan Nagar, Chengalpattu, Tamil Nadu; 4 Central Research Laboratory, Meenakshi Ammal Dental College and Hospital, Chennai, Tamil Nadu, India; 5 DBT-BIF Centre, PG & Research Department of Biotechnology & Bioinformatics, Holy Cross College (Autonomous), Trichy, Tamilnadu; 6 Department of Biochemistry, Panimalar Medical College Hospital & Research Institute, Varadharajapuram, Poonamallee, Chennai-600 123, Tamil Nadu, India

**Keywords:** Colon cancer, importin-11, molecular models, molecular docking

## Abstract

Colorectal cancer (CRC) is the most familiar malignancy worldwide. Hence, searching for novel therapeutic options is of highest priority. Therefore, it is of interest to design inhibitors to the protein target importin-11, which transports β-catenin linked to colon cancer cells. However, the structure of importin-11 is not known. Hence, we use a homology model of importin-11 to dock potential interactions with five phyto compounds using molecular interaction features for further consideration.

## Background

Compared to all cancer types, colorectal cancer is most frequent form of cancer in oncologic pathology [1]. At present, it is one of the main malignant cancers in the gastrointestinal tract, its shows 13% of all malignant tumors, so it is second most frequent cause of death related to cancer affecting men as women in the equal manner worldwide, developed and undeveloped Countries, and also it is estimated to overcome the death rate of heart diseases in the coming years [2- [4].The rate of colon cancer was extremely high in developed countries (North America, Australia, and New Zealand) than developing countries [5].In case of India, (AARs) has been estimated to be 4.4 and 3.9 /100,000 for males and females, respectively (National Cancer Registry Program, ICMR, 2014).According to the data available on Madras Metropolitan Tumour Registry (MMTR) as on 2012–2013, colorectal cancer has crude incidence rate (CIR) of 8.2 per 100,000. Results of these reports clearly indicated that colon cancer is one major health problem in human. Hence there is an urgent need to find out new molecules to combat cancer with novel mechanism of action. Multidisciplinary scientific investigations are creates greatest efforts to battle cancer, but the sure-shot, just right cure is yet to be brought into world of medicine. Any useful solution in fighting this alarming disease is of paramount importance. Another source of anticancer drugs is natural products, which normally seem to be more effective and/or less toxic. Approximately 80% of colorectal cancers are related with mutations in a gene called APC that result in high levels of the β-catenin protein. This raise in β-catenin is followed by the protein's accumulation in the cell nucleus, where it can trigger numerous genes that drive cell proliferation and support the development and maintenance of colorectal tumors. However how β-catenin go into the cell nucleus following its levels rise is poorly understood. Mis et al. (2019)[6] screened the human genome used for genes that maintain β-catenin's activity in colorectal cancer cells following its levels has been elevated by mutations in APC and identified IPO11 as a necessary factor or β-catenin-mediated transcription in APC mutant CRC cells. The researchers also identified that Importin-11 binds to β-catenin and accompany it into the nucleus of colorectal cancer cells through mutations in APC. Inhibiting the activity of Importin-11 from these cells prohibited β-catenin from entering the nucleus and activating its target genes. Mis et al. (2019) also revealed that Importin-11 levels are frequently elevated in human colorectal cancers. Furthermore, inhibiting the activity of Importin-11 suppressed the growth of tumors formed by APC mutant cancer cells isolated from patients [6].There are many reports also showed that Importin-11 is one of main transport receptors and act as target for many cancer types. Scott et al. 2000 [7] also studied that importin-11 as a new member of the karyopherin family of transport receptors, and identify UbcM2 as a nuclear member of the E2 ubiquitin-conjugating enzyme family. Chen et al. (2017)[8] studied that Importin-11 traffics the tumor suppressor PTEN into the nucleus and in so doing protects it from cytoplasmic proteins that cause PTEN degradation.Chen et al.[8] provide strong evidence that the importin β family member Importin-11 (IPO11) binds to PTEN and transports it into the nucleus. In prostate cancer cells lacking IPO11 function, PTEN was found to be much more cytoplasmic at stable state than control cells and in dynamic experiments IPO11 disturbance greatly slowed down entry of PTEN into the nucleus. Therefore, it is of interest to describe the design of inhibitors to the protein target importin-11 which transports β-catenin linked to colon cancer cells (Table 1).

## Materials and Methods:

### Template search and Sequence Alignment:

In the current examination, the protein sequence of of Importin-11 was retrieved from Uni-protKB/Swissprot database (ID Q9UI26)[9].The protein sequence consisted of 975 amino acids. A BLASTP [10] search with default parameter was executed against the use RCSB PDB [11] to discover template for homology modelling.Based on the highest uniqueness with high score and lesser e-value, Importin beta-like protein KAP120 [Saccharomyces cerevisiae S288C (PDB ID: 6FVB) which had the Sequence identity of 27% was selected as template structure for modeling. Clustal W program was used for sequence arrangement among the target and template [12].

### 3D structure generation:

The details accuiquired from sequence alignment was helped to build the 3D model of Importin-11 protein using educational edition of MODELLER (http//:www.salilab. org/modeller) [13].Five 3D models were built and visualized by PyMol software [14].The best model was graded based on discrete optimized protein energy (DOPE) score produced by MODELER software.

### Assessment of the homology model

With the intention to evaluate the consistency of the Importin-11 modelled structure, structural analysis and verification server (SAVES) (http://servicesn.mbi.ucla.edu/SAVES/) was used. The phi (Φ) and psi (Ψ) torsion angles of protein was calculated using PROCHECK program, as firmed by Ramachandran plot statistics [15]. The root mean square deviation (RMSD) was computed by structural superimposition of template (6FVB) and predicted structure of Importin-11 for the consistency of the model using UCSF Chimera [16].

### Molecular docking:

#### Ligand retrieval:

The structures Thalicarpine (CID:21470) Tylocrebrine (CID: 246845) Emetine(CID: 10219) Kamebanin(CID: 12004580) Helenalin(CID: 23205) were downloaded from the PubChem database. For docking studies, these downloaded compounds structures were used. All the compounds were downloaded in SDF format and it was converted as PDB format using Online Smiles Translator.

#### Prominent binding site prediction:

Before to docking analysis, important active site of modeled Importin-11 protein was identified using CASTp server [17] Key binding pockets were identified for analysis of active binding amino acids residues and comparison of the docking results.

#### Docking analysis:

Molecular docking studies were carried out using Patch Dock server [18].Both protein and compounds were uploaded as PDB format in Patch dock server for docking studies. LigPlot was used to analyses the docking solutions and confirmation obtained from PatchDock.

### Analysis of drug likeness of selected compounds:

The drug likeness calculation of Thalicarpine, Tylocrebrine, Emetine, Kamebanin and Helenalin was performed by Lipinski filter (http://www.scfbio-iitd.res.in/software/drugdesign/ lipinski.jsp), according to which an orally active drug should obey at least of four of the five laid down criterion for drug likeness namely: molecular mass, cLogP, hydrogen donor and acceptor and molar refractive index [19].

## Results and discussion:

**Figure 1:**
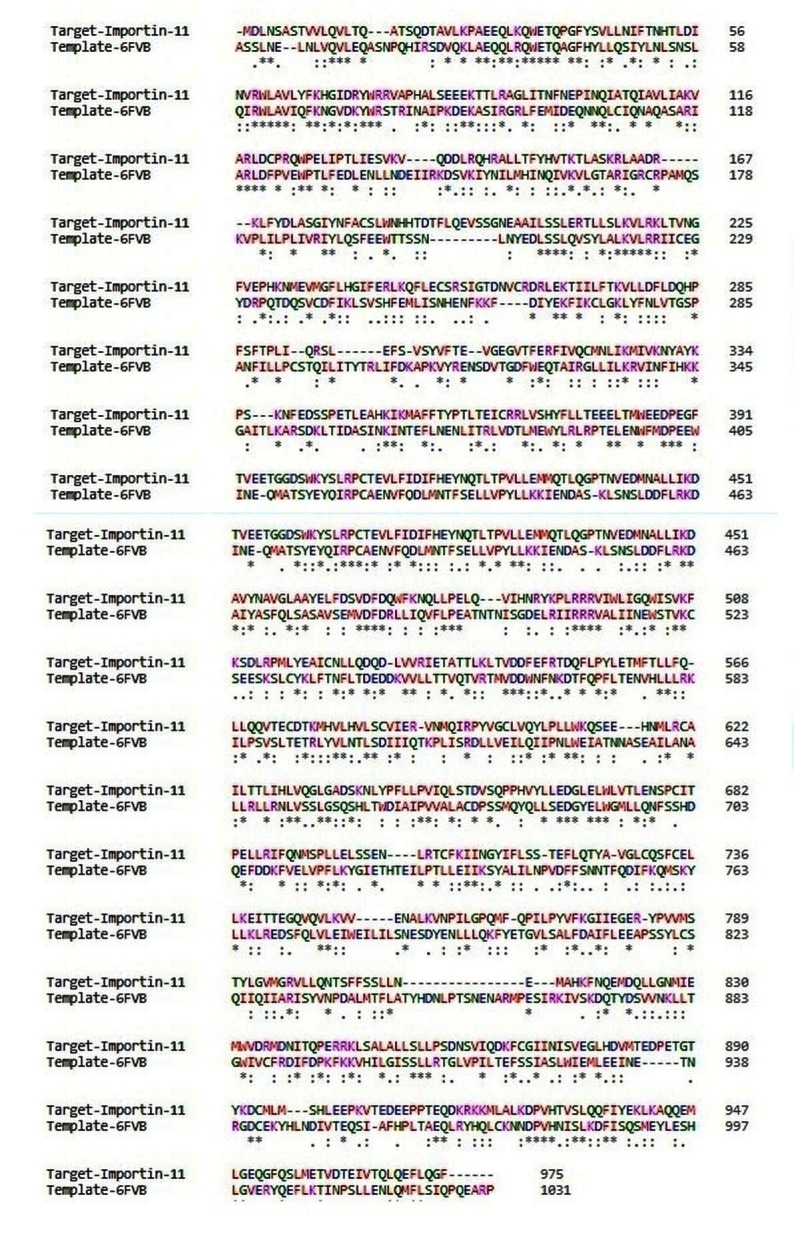
Sequence alignment of Importin-11protein (Target) with 6FVB (Template) created by the ClustalW algorithm. In the sequences, an asterisk (*) indicated a matching or conserved residue, a colon (:) indicated a preserved replacement, a stop (.) indicated a partially conserved substitution.

**Figure 2:**
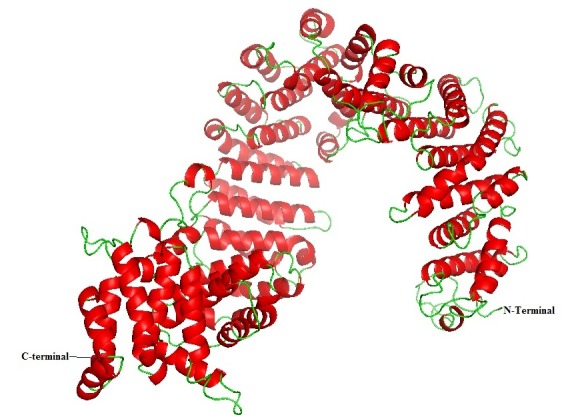
The best modeled structure of Importin-11protein obtained from Modeller 9v10. Red colour indicates alpha helices, yellow colour indicates the beta sheets and green colour indicate the loops.

**Figure 3:**
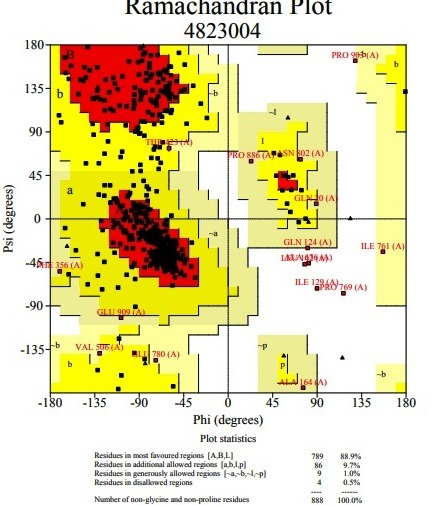
Ramachandran plot of the developed homology model of Importin-11protein. The most favored regions are colored red; additional allowed, generously allowed and disallowed regions are shown as yellow, light yellow and white fields, respectively.

**Figure 4:**
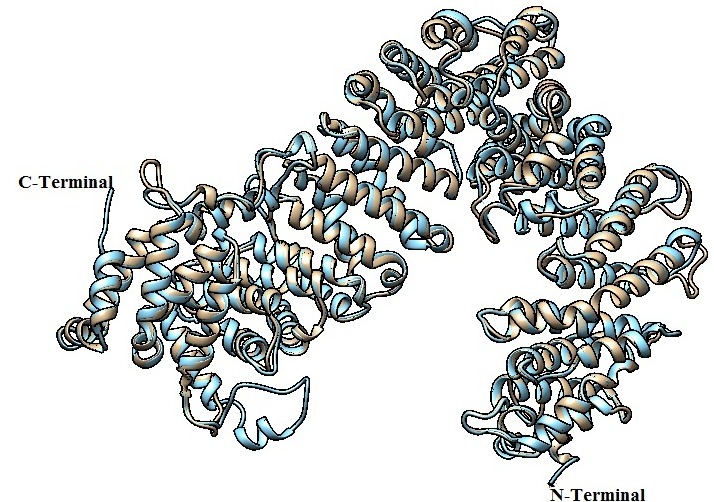
Superimposed structure of Importin-11protein with template structure. White colour indicates target and light blue indicate template.

The crystallographic structure of Importin-11 was not found in the PDB, however the sequence similarity between Importin beta-like protein KAP120 [Saccharomyces cerevisiae S288C (PDB ID: 6FVB) and Importin-11 (swissprot id: Q9UI26) was 27% which in turn has made it a reasonable template for modeling a three dimensional structure of Importin-11. (Figure 1) showed the sequence alignment of the Importin-11 protein with the similar template protein that was needed for homology modelling. MODELLER 9v10 was used to create the structure of target Importin-11 based on the 6FVB as template. The 3D structure provided important approaching into molecular function. The input parameters of MODELLER were set to create five models by the level of elevated optimization and fulfilling spatial restraints in terms of protein density function. The final model (Figure 2) was chosen based on low MODELLER objective function and the high value of negative DOPE potential. The quality of the concluding model was assessed by PROCHECK14, which showed the in depth residue-by-residue stereo chemical value of the predicted model and that was discovered to be good. The Phi/ Psi distribution of the Ramachandran plot for Importin-11 have shown 88.9% of the residues in the core region or most favourable regions, 9.7 % residues in the additionally allowed regions, 1.0 % in the generously allowed regions and 0.5 % in disallowed regions (Figure 3). The RMSD value of constructed 3D model has similar to their related template structure (0.294 Å) (Figure 4).These validation processes showed that backbone conformations and non-bonded interactions were in the limits, hence it was recognized as reliable structure and it can be used for additional analysis.

**Figure 5:**
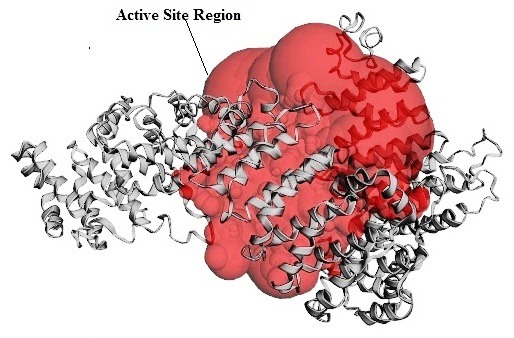
Predicted active site region usingm CASTp server

In order to identify the binding conformation of selected five compounds with Importin-11 molecular docking studies was carried out. Docking of selected five compounds with the target protein of Importin-11 was carried out with respect to interacting amino acids, ligand and protein atoms participated in hydrogen bonding, and docking score with ACE area (Table 2). Analysis of docking results clearly showed that many amino acids residues involved in H bonding interaction were found to be in the major active site residues identified through CASTp server (Figure 5). LigPlot analysis was also performed to elucidate the interaction between the selected compounds and Importin-11 that assisted in understanding hydrophobic interactions as well as hydrogen-bonding patterns. Presence of H- bond interaction helped the docked complex to attain a recognized conformation. Hence the results of this docking study showed that selected five phyto compounds had good Hbond interaction with Importin-11. So this study suggested that, these phyto compounds could inhibit of activity of Importin-11 protein and acts as best anticancer agents.The drug likeness properties of selected five compounds were predicted by Lipinski filter and the properties of the ligands with respect to calculation of adsorption, distribution, metabolism, excretion and toxicity. In the present study selected all the five compounds had good drug likeness properties, so it used as drug for application in biological systems (Table 3).

## Conclusions:

We report the modeling docking analysis of importin-11 homology model with five phyto compounds in the context of colorectal cancer for further consideration.

## Figures and Tables

**Table 1:** List of selected phytocompounds:

S. No	Compound Name	Source of Plant
1	Thalicarpine	Thalictrum dasycarpum Fisch
2	Tylocrebrine	Tylophora crebriflora Blake
3	Emetine	Cephaelis acuminate Karst
4	Kamebanin	Rabdosia umbrosa var.
5	Helenalin	Heliotropium indicum L.

**Table 2:** Patch dock results of Importin-11 protein with selected compounds:

Compounds	Patch dock score (kca l/mol)	ACE	Atomic interaction	H-bond donor	H-bond acceptor	H-bond distance
Thalicarpine	4156	-85.39	LYS 757 NH-O	PR(H)-O	Li(O)	2.66
			LYS 919 NH-O	PR(H)-O	Li(O)	2.43
Tylocrebrine	5278	-337.74	GLY 310 H-O	PR(H)-O	Li(O)	1.8
Emetine	6132	-139.32	SER 404 O-H	Li(H)-O	Li(H)	2.62
Kamebanin	4340	106.59	LYS 757 NH-O	PR(H)-O	Li(O)	1.78
Helenalin	3970	2.88	HIS 929 NH-O	PR(H)-O	Li(O)	2.71

**Table 3:** The drug likeness properties of selected compounds.

Compound name	Molecular Mass^a^	Hydrogen bond donor^b^	Hydrogen bond donor^c^	LOGP^d^	Molar Refractivity^e^
Thalicarpine	696	0	10	5.30418	190.491333
Tylocrebrine	393	0	5	4.315198	113.86496
Emetine	480	1	6	4.329039	136.066696
Kamebanin	334	3	4	2.0668	90.043365
Helenalin	262	1	4	1.2463	68.041779
^a^Molecular mass less than 500 Dalton;^b^High lipophilicity (expressed as LogP less than 5);^c^Less than 5 hydrogen bond donors;^d^Less than 10 hydrogen bond acceptors.
